# Periodontal disease severity is associated to pathogenic consortia comprising putative and candidate periodontal pathogens ^*^

**DOI:** 10.1590/1678-7757-2022-0359

**Published:** 2023-01-06

**Authors:** Lélia Lima ARAÚJO, Talita Gomes Baêta LOURENÇO, Ana Paula Vieira COLOMBO

**Affiliations:** 1 Universidade Federal do Rio de Janeiro Faculdade de Odontologia Programa de Pós-Graduação em Odontologia Rio de Janeiro Brasil Universidade Federal do Rio de Janeiro, Faculdade de Odontologia, Programa de Pós-Graduação em Odontologia (Periodontia), Rio de Janeiro, Brasil.; 2 Universidade Federal do Rio de Janeiro Instituto de Microbiologia Departamento de Microbiologia Médica Rio de Janeiro Brasil Universidade Federal do Rio de Janeiro, Instituto de Microbiologia, Departamento de Microbiologia Médica, Rio de Janeiro, Brasil.

**Keywords:** Gingivitis, Periodontitis, Dental plaque, PCR, Microbial consortia

## Abstract

Based on a holistic concept of polymicrobial etiology, we have hypothesized that putative and candidate periodontal pathogens are more frequently detected in consortia than alone in advanced forms of periodontal diseases (PD).

**Objective:**

To correlate specific consortia of periodontal pathogens with clinical periodontal status and severity of periodontitis.

**Methodology:**

Subgingival biofilm was obtained from individuals with periodontal health (113, PH), gingivitis (91, G), and periodontitis (209, P). Genomic DNA was purified and the species *Aggregatibacter actinomycetemcomitans* (Aa), Aa JP2-like strain, *Porphyromonas gingivalis* (Pg), *Dialister pneumosintes* (Dp), and *Filifactor alocis* (Fa) were detected by PCR. Configural frequency and logistic regression analyses were performed to correlate microbial consortia and PD.

**Results:**

Aa + Pg in the presence of Dp (phi=0.240; χ^2^=11.9, p<0.01), as well as Aa JP2 + Dp + Fa (phi=0.186, χ^2^=4.6, p<0.05) were significantly more associated in advanced stages of P. The consortium Aa + Fa + Dp was strongly associated with deep pocketing and inflammation (p<0.001). The best predictors of disease severity (80% accuracy) included older age (OR 1.11 [95% CI 1.07 – 1.15], p<0.001), Black/African-American ancestry (OR 1.89 [95% CI 1.19 – 2.99], p=0.007), and high frequency of Aa + Pg + Dp (OR 3.04 [95% CI 1.49 – 6.22], p=0.002).

**Conclusion:**

Specific microbial consortia of putative and novel periodontal pathogens, associated with demographic parameters, correlate with severe periodontitis, supporting the multifactorial nature of PD.

## Introduction

Periodontal disease (PD) is a biofilm-driven chronic inflammatory condition that eventually leads to the destruction of the supporting periodontal tissues. Such disease results from an imbalance of the dynamic interaction between the microbiota and the host response, modulated by environmental, systemic, and genetic factors.^1 , 2^ Shifts in the oral microbiome are implicated in the onset and/or progression of PDs,^3 , 4^ and although over 400 species colonize the subgingival biofilm,^5 , 6^ only a fraction of them has been implicated in destructive diseases.^7 - 9^ Thus, PDs are not the “one pathogen, one infection” type of disease, since they are associated with an unbalanced consortia of members of the normal microbiota, including pathobionts and commensals.^2 , 3 , 10^ Advances in biotechnology for more rapid and accurate identification of microorganisms in oral specimens have provided insights about the role of specific bacteria within microbial complexes in the etiology and pathogenesis of severe form of periodontitis.^11^ Although *Aggregatibacter actinomycetemcomitans* ( *Aa* ) and *Porphyromonas gingivalis* are the main cultivable putative periodontal pathogens recognized worldwide,^12 , 13^ novel taxa are currently being considered as potential periodontal pathobionts.^14 - 16^ For instance, evidence suggests strong associations between *Filifactor alocis, Dialister pneumosintes,* and even uncultivated taxa of *Tannerella* and *Saccharibacteria* with advanced forms of periodontitis and peri-implantitis.^15 - 20^ The potential mechanisms of interactions among members of the oral microbiota and between these communities and the periodontal environment are endless and not fully understood. As previously described for the microbial complexes of the subgingival plaque,^8^ screening for combinations of novel potential pathogens and their correlation with periodontal destruction and inflammation may provide additional information regarding other microbial consortia as predictors of severe forms of disease. Therefore, given the dynamic polymicrobial nature of PD etiology, this study aimed to evaluate whether specific putative and novel periodontal pathogens are more frequently detected combined or as isolated species in severe forms of PD.

## Methodology

### Ethical statement

This single-center, observational cross-sectional study was conducted in full accordance with the 1964 Helsinki Declaration and its later amendments. The study protocol was approved by the Human Research Ethics Committee of the Clementino Fraga Filho Hospital of the Federal University of Rio de Janeiro (UFRJ), Brazil (#3.792.702). To participate, individuals were informed about the risks and benefits of the study and signed an informed consent form. The respective manuscript was written following the Strengthening the Reporting of Observational Studies in Epidemiology (STROBE) guidelines.

### Sample population

Individuals who sought dental treatment at the Dental School of the UFRJ until December/2019 were selected for the study. Subjects had to be ≥18 years old and present at least 18 teeth. Exclusion criteria were presence of chronic inflammatory systemic diseases, use of topical or systemic antimicrobials in the last six months, use of anti-inflammatory drugs in the last three months, periodontal therapy in the last six months, need for antibiotic prophylaxis, ongoing orthodontic treatment, pregnancy, or nursing. Participants were submitted to a medical and dental anamnesis and were asked to fill out a questionnaire providing demographic and lifestyle data. Smoking was recorded as never having smoked and current or former smokers (smoking cessation <10 years). Sample size calculation was based on the frequency of detection of the four pathogens ( *Aa, D. pneumosintes, F. alocis, P. gingivalis* ) in subgingival biofilm of individuals with periodontal health and diseases from our previous data.^19^ The program PS: Power and Sample Size Calculation, version 3.1.6, was used to estimate the sample size of 41 patients per group, considering a precision level of 5%, a statistical power of 90%, and differences in prevalence of these species varying from 33-54% between groups.

### Clinical examination and periodontal diagnosis

Periodontal clinical measurements were performed at six sites per tooth, excepting third molars, with a standard periodontal probe (UNC-15, Hu-Friedy, Chicago, IL, USA) by trained and calibrated Periodontists (overall intra-examiner ICC >0.85 for probing pocket depth [PPD]). Parameters measured included PPD, clinical attachment level (CAL), presence or absence of supragingival biofilm (PL), gingival marginal bleeding (GI), bleeding on probing (BOP), calculus (CA), and suppuration (SUP). Eligible individuals were diagnosed as presenting periodontal health (PH), gingivitis (G), or periodontitis (P), according to Da Silva-Boghossian, et al.^21^ (2011). Briefly, PH was defined as <10% of sites with BOP, no PPD, or CAL >3 mm, although PPD or CAL =4 mm in up to 5% of the sites without BOP was considered. G was defined as ≥10% of bleeding sites (BOP or GI) with PD≤3 mm, on an intact or reduced/stable periodontium in a patient without history of periodontitis. P was defined as ≥10% of teeth with PPD and CAL≥5 mm, or ≥15% of teeth with PPD and CAL≥4 mm, with >10% of sites presenting BOP. Periodontitis patients were further classified by disease severity according to the current periodontal disease classification guidelines^22^ . Patients with any other dental needs were referred for treatment at the same institution and instructed to proper home care procedures.

### Subgingival biofilm sampling

In patients with PH, subgingival biofilm samples were obtained from 7 to 14 healthy sites (PPD <4 mm, no bleeding); in patients with gingivitis, from 7 to 14 bleeding sites; and in patients with periodontitis, from 7 to 14 periodontal lesions. The selected sites were isolated, air dried, and supragingival plaque removed with sterile gauze. Then, subgingival biofilm samples were taken using sterile Gracey curettes and scalers (Hu-Friedy) and pooled into individual microtubes containing 300 µl of Tris-EDTA buffer (10 mM Tris-HCl, 1 mM EDTA, pH 7.6; Sigma-Aldrich Brasil Ltda, SP, Brazil). Samples were maintained on ice for transportation to the laboratory and kept at – 25ºC for DNA extraction within 24h.

### Bacterial DNA from subgingival biofilm

Extraction and purification of bacterial DNA from subgingival biofilm samples were conducted according to a commercial system (MasterPure DNA Purification Kit, Epicentre, Madison, WI, USA). Concentration and purity of DNA samples were evaluated by agarose gel electrophoresis and spectrophotometry (Nano Drop Lite^™^, Thermo Fisher Scientific, Wilmington, DE, USA). All samples with low concentrations or negative results were evaluated for presence of bacterial DNA by amplifying the 16S rRNA gene, as described below.

### Detection of putative and potential periodontal pathogens

PCR amplification of target sequences was performed using two sets of primers to each bacterial species and one set of universal primers for the 16S rRNA gene (Supplementary Table 1 ). Amplifications were performed for each species, in a mix reaction of 25 μL using a commercial kit (GoTaq^®^ Green Master Mix, Promega Biotecnologia do Brasil Ltda., SP, Brazil). Different primer concentrations were used for distinct species: 2 pmol/μL for universal primers, 1 pmol/μL for *P. gingivalis* primers, 0.4 pmol/μL for *Aa* ( *Aa* JP2), and 0.2 pmol/μL for *D. pneumosintes* and *F. alocis* primers. Negative (no DNA template in Rnase-free water) and positive controls were added to each run. For positive controls, 100 ng/µl of DNA obtained from reference strains were used: *A. actinomycetemcomitans* (ATCC 29523), *D. pneumosintes* (ATCC 33048), *F. alocis* (ATCC 35896), and *P. gingivalis* (ATCC 33277). The highly leukotoxic *Aa* JP2 HK921 strain was gently donated by Dr. Dorte Haubek of the University of Aarhus, Denmark. Amplification protocols were performed in a thermocycler (T100^™^ Thermal Cycler, BioRad Laboratories Inc., Hercules, CA, USA), as described in supplementary Table 1 . PCR products were analyzed on a DNA dye-stained (SBYR safe Invitrogen, SP, Brazil) 1% agarose gel electrophoresis (UltraPure Agarose, Gibco-BRL, Grand Island, NY, USA), and visualized on a capture-imaging system (MiniBis Pro,Bio-Imaging Systems^®^, Jerusalem, Israel). A 100 bp ladder was used as a standard molecular weight (Sinapse^®^ Inc., SP, Brazil).

**Table 1:** Socio-demographic data of the study population

Parameters	Periodontal Health (n = 113)	Gingivitis (n = 91)	Periodontitis (n = 209)
Mean (SD) Age (years)*	27.2 (10.2)	30.1 (12.6)	43.6 (13.0)
Gender (%)			
Females	68.1	70.3	60.3
Males	31.9	39.7	39.7
Race (%)†			
White	69.0‡	46.2	39.3
African-American	8.0	17.6	25.4
Others	23.0	36.3	35.3
Education (%)†			
Middle School	1.8	5.6	22.9
High School	8.8	50.6	39.9
Higher Education	89.4‡	43.8	37.2
Monthly family income (%)†			
≤ 2 minimum wage	18.0	52.8	66.7
> 2 minimum wage	82.0	47.2	33.3
Smoking (%)†			
Non-smokers	93.8	90.1	83.7
Former smokers	4.4	1.1	13.4
Current smokers	1.8	8.8	2.9

Symbols refer to significant differences among groups (*Kruskal-Wallis and †χ^2^ tests, p<0.05).

### Statistical analysis

Descriptive statistics of socio-demographic and clinical measurements were computed for each subject and then averaged across subjects within groups. Differences on these parameters among groups were sought by using Kruskal-Wallis, Mann-Whitney, and χ^2^ tests. For microbial analysis, 2×2 contingency tables were constructed to determine the frequency distribution of each species regarding clinical status, disease severity, inflammation, and socio-demographic features (χ^2^ tests). Correlations between pairs and trios of bacteria and between these combinations and clinical conditions were investigated by constructing multiway contingency tables, evaluated by using configural frequency analysis, phi, and χ^2^ coefficients. Finally, multivariate logistic regression analysis was conducted to determine the best microbial consortia and non-microbial parameters as predictors of periodontitis and/or disease severity. The stepwise Wald method was selected and ORs with 95% CI were reported. The significance level determined was 5%. All analyses were performed using a commercial software package (IBM SPSS^®^ Statistics, IBM Brasil, SP, Brazil).

## Results

### Clinical and socio-demographic features of the study population

**Figure 1:**
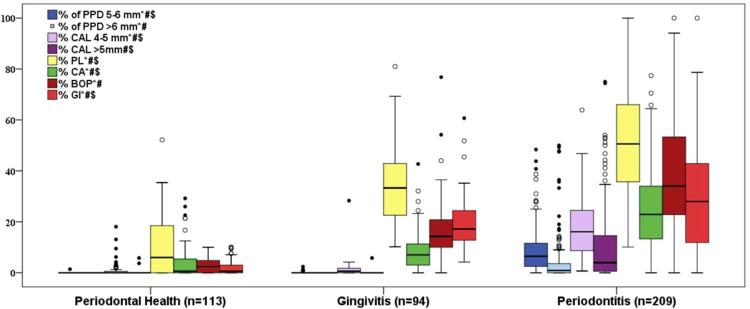
Clinical data of the study population (n=413). Box plots represent the distribution in quartiles of mean % of sites with probing pocket depth (PPD) of 5-6 mm; PPD >6 mm; clinical attachment level (CAL) of 4-5 mm; CAL >5 mm; visible supragingival plaque (PL), marginal gingival bleeding (GI), bleeding on probing (BOP) and calculus (CA). Whiskers indicate minimum and maximum values; circles indicate outliers. All parameters differed significantly among groups (Kruskal-Wallis test, p<0.05). *Refers to significant differences between periodontal health and gingivitis; #between gingivitis and periodontitis and $between periodontal health and periodontitis groups (Mann-Whitney test, p<0.05).

Table 1 and Figure 1 show the characteristics of the 413 eligible participants of the study, classified in three clinical groups. Patients with P were significantly older, current or former smokers, and of lower socioeconomic and schooling level status than individuals in the other groups (p<0.05; χ^2^ test). Almost 70% of diseased patients had advanced forms of the disease (stages III and IV), and almost 90% presented a more localized form of PD^22^ . Among those, patients with severe diseases were older (47±11 years) and presented lower schooling level (27.3% in middle school) compared to patients with light to moderate disease (age: 35.8±12 years; 13% in middle school p<0.05; data not shown).

### Frequency of detection of putative and candidate periodontal pathogens

**Figure 2:**
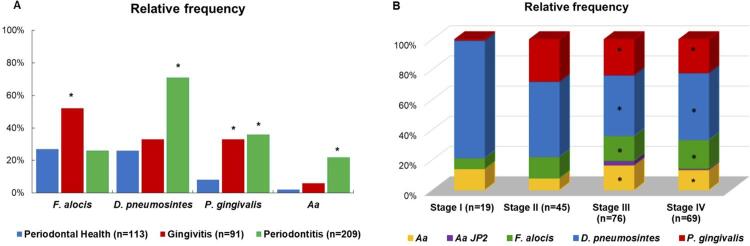
Prevalence of periodontal pathogens in the subgingival biofilm. (A) Bars represent the relative frequency (%) of putative and potential novel periodontal pathogens across the periodontal clinical groups. (B) Columns represent the % of pathogens in subgingival biofilm of 209 individuals with periodontitis according to stages of disease severity. *Aggregatibacter actinomycetemcomitans* (Aa); Aa JP2: highly leukotoxic strain of *A. actinomycetemcomitans* . *Significant differences between disease and health; χ^2^ test; p<0.05.

The putative periodontal pathogens *Aa* and *P. gingivalis* and the species *D. pneumosintes* were significantly more prevalent in periodontal diseases than health, whereas *F. alocis* predominated in G group (p<0.05, χ^2^ test; Figure 2A ). Among P patients, however, *F. alocis* was significantly more increased in advanced stages of disease (p<0.05, χ^2^ test; Figure 2B ). The highly leukotoxic strain *Aa* JP2 was detected in only six patients, one with G and five with P stages III/IV.

Due to differences in socio-demographic features among clinical groups, associations between periodontal bacteria and these parameters were explored for further adjustments (Supplementary Table 2 ). We failed to observe significant differences in the distribution of these species alone or combined for gender or smoking status. *D. pneumosintes* was the only species significantly more prevalent in older individuals (>34 years). Of the 6 *Aa* JP2-positive patients, five were African Americans. *P. gingivalis, Aa* , and *D. pneumosintes* were more prevalent in individuals with lower socioeconomic status (p<0.05; χ^2^ test). Nevertheless, when we remove individuals with low income or schooling level from the analysis, the prevalence of these species in the clinical groups present a similar distribution observed in the whole sample (data not shown).

**Table 2:** Stepwise regression analysis (backward Wald method) using sociodemographic parameters and microbial consortia as predictors of disease severity (mild disease = stages I and II; advanced disease = stages III and IV of periodontitis)

Predictor variables	β	SE of β	Wald χ^2^ test	p	Odds ratio	95% CI of OR	
						Lower	Upper
Constant	-4,655	0.889	27.41	<0.001	0.01		
Age	0.107	0.018	35.02	<0.001	1.11	1.07	1.15
Race (African-american heritage)	0.636	0.236	7.29	0.007	1.89	1.19	2.99
Aa + *P. gingivalis* + *D. pneumosintes*	1.113	0.365	9.29	0.002	3.04	1.49	6.22

Omnibus Tests of Model Coefficients: −2 Log likelihood = 166.93; Cox & Snell R^2^ = 0.288; Nagelkerke R^2^ = 0.404. Overall χ^2^ test for the model 62.41, 3 df, p<0.001.

Variable(s) entered: age, skin color/ethnicity, schooling level, smoking, income, gender, Aa + *P. gingivalis* + *F. alocis* ; Aa + *D. pneumosintes* + *P. gingivalis* ; Aa + *F. alocis* + *D. pneumosintes* ; *P. gingivalis* + *F. alocis* + *D. pneumosintes* ; *P. gingivalis* + *F. alocis* + AaJP2; *F. alocis* + *D. pneumosintes* + AaJP2; *P. gingivalis* + *D. pneumosintes* + AaJP2.

### Associations among periodontal species within consortia

**Figure 3:**
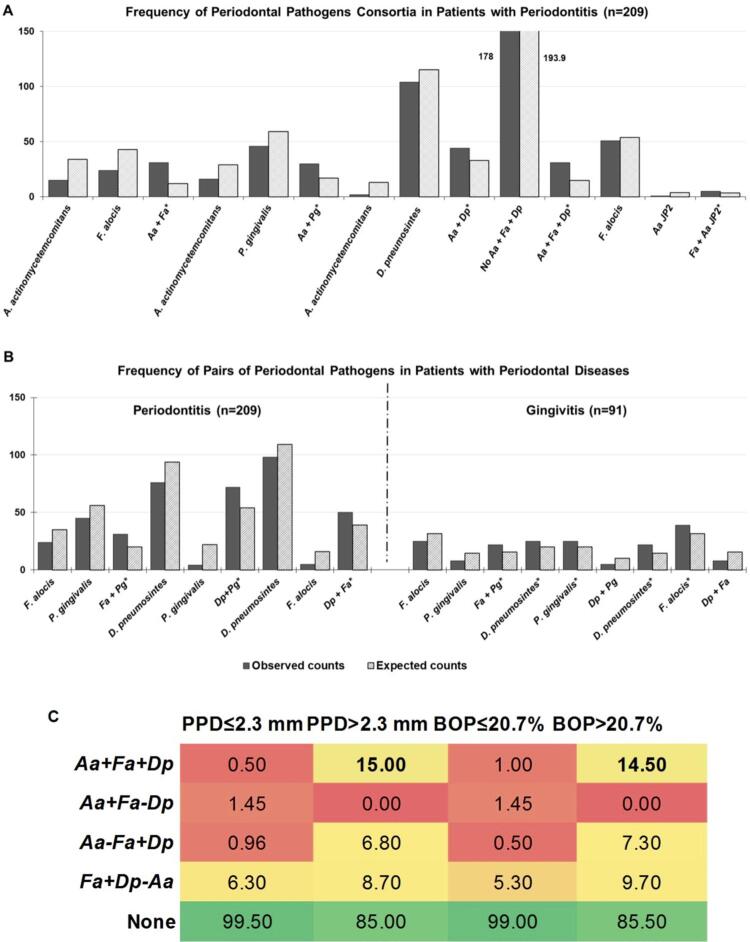
Periodontal consortia associated with periodontal diseases. Columns represent observed (dark) and expected (dashed) counts of different bacteria combined and as single species in subgingival biofilm samples. (A) Consortia exclusively predominant in patients with periodontitis (n=209). (B) F. alocis (Fa) + P. gingivalis (Pg) were frequently detected in both gingivitis (n=91) and periodontitis samples. The combination of D. pneumosintes (Dp) + F. alocis (Fa) was rarely detected in gingivitis, but frequently observed in periodontitis. *Significant differences between observed and expected counts (χ² test; p<0.05). (C) The frequency (%) of periodontal bacteria in combinations was computed for 413 individuals presenting probing pocket depth (PPD) and bleeding on probing (BOP) > and ≤ the median values (PPD=2.27 mm; % sites BOP=20.7). The consortia Aa + Fa + Dp was the only one significantly more prevalent in patients with high number of deep pockets (χ^2^=37, phi=0.501, p<0.001) and bleeding sites (χ²=31, phi=0.458, p<0.001) compared to healthier individuals.

Since only a few patients presented simultaneously >3 of the species evaluated in the subgingival biofilm sample, analyses were limited to consortia composed by two or three different species. Associations among species were first examined by comparing the expected and observed frequencies of microorganisms in combination or as single isolates in each clinical condition. For the PH group, we failed to observe significant differences in the frequency of detection between single and combined species. However, the strongest associations of microbial pairs in P patients were seen for *Aa + F. alocis* (phi=0.498; χ^2^=51.3), *Aa* + *P. gingivalis* (phi=0.319; χ^2^=21.2) and *Aa* + *D. pneumosintes* (phi=0.290; χ^2^=17.6; p<0.001). Among combinations with three periodontal species, the consortia *Aa* + *F. alocis* + *D. pneumosintes* (phi=0.504; χ^2^=37.6; p<0.001) was the most strongly associated with P ( Figure 3A ). Although small, the only association with the *Aa* JP2 clone in P was with *F. alocis* (phi=0.191, χ^2^=7.6; p=0.006). Moreover, *F. alocis* and *P. gingivalis* were significantly correlated in both G (phi=0.304; χ^2^=8.4; p=0.004) and P (phi=0.248; χ^2^=13; p<0.01) samples; whereas *D. pneumosintes* + *P. gingivalis* or + *F. alocis* were inversely associated in gingivitis, but commonly detected together in P ( Figure 3B ). When analyzing these consortia within periodontitis samples, *Aa* + *P. gingivalis* in the presence of *D. pneumosintes* (phi=0.240; χ^2^=11.9, p<0.01) was predominant in advanced stages of P. Likewise, *Aa* JP2 clone + *D. pneumosintes* in the presence of *F. alocis* was modestly correlated in stages III and IV of P, but not in initial stages of the disease (phi=0.186, χ^2^=4.6; p<0.05; data not shown).

### Relation between consortia and clinical parameters

The consortia of bacteria were associated with clinical parameters of disease by computing their frequency in the 413 individuals presenting PPD and BOP > and ≤ the median values (PPD=2.27 mm; % sites BOP=20.7). In individuals with high number of deep pockets and bleeding sites ( Figure 3C ), only the consortium *Aa + F. alocis* + *D. pneumosintes* was more frequently found in combination than single species (PPD: phi=0.501, χ^2^=37; BOP: phi=0.458, χ^2^=31; p<0.001). Increased age was also correlated with 2 dual-species consortia: *D. pneumosintes* + *F. alocis* (20%) and *D. pneumosintes* + *P. gingivalis* (27.5%) compared to patients with ≤34 years of age (10.8% and 12.2%, respectively, p<0.01; data not shown).

### Determining disease severity by novel and putative periodontal pathogenic consortia

Socio-demographic parameters and periodontal pathogens, alone and combined, were used in univariate and multivariate logistic regression analyses as potential discriminators of disease severity. The best fitting model, with 80% of accuracy (χ^2^ (df 3)=166.93; p<0.001; Nagel R^2^=0.404), to discriminate severe periodontitis in relation to mild to moderate disease ( Table 2 ) included increased age (OR 1.11 [95% CI 1.07 – 1.15], p<0.001), Black/African American heritage (OR 1.89 [95% CI 1.19 – 2.99], p=0.007, and high frequency of *Aa* + *P. gingivalis* + *D. pneumosintes* (OR 3.04 [95% CI 1.49 – 6.22], p=0.002).

## Discussion

Our current comprehension about PD’s etiology and pathogenesis is based on a holistic concept of polymicrobial synergy and dysbiosis of the periodontal biofilm in a particular host and environment.^2 , 10^ The multi-species periodontal biofilm comprises a large variety of microorganisms, including recognized commensals and pathobionts, as well as numerous unknown taxa.^23^ Despite tremendous progress on biofilm research, the complex interspecies dynamics that occur within the gingival/subgingival biofilm in a condition of health and disease is not fully understood.^24^ Deciphering complex interactions among several microorganisms within this structure may begin by looking at the frequency of detection of specific consortia in distinct oral status. Therefore, we hypothesized that patients with periodontitis, especially with more advanced forms, presented higher prevalence of putative and novel periodontal pathogens in association (consortia) than as single species in the subgingival biofilm compared to periodontally healthy individuals. For that, we focused on two major periodontal pathogens, *Aa* (including the JP2-like clone) and *P. gingivalis* , and potential novel pathogens currently related to advanced forms of disease, *D. pneumosintes* and *F. alocis* . Given the limited number of target species evaluated in biofilm samples, well stablished PCR protocols were chosen as the microbial identification method (suppl. table 1 ). As expected, *Aa, P. gingivalis* , and *D. pneumosintes* were more prevalent in individuals with periodontitis than in healthy patients.^8 , 12 , 15 , 18 - 20 , 25 - 27^ However, the high frequency of *F. alocis* in G group compared to PH and P groups was surprising. Such species is highly prevalent in periodontitis, peri-implantitis, and endodontic infections, and its high values in our G patients compared to P disagrees with data reported by other authors.^9 , 28^ On the other hand, *F. alocis* has been strongly associated with bleeding periodontal sites^29^ and gingivitis in diabetic pregnant women.^30^ This bacteria can also induce secretion of pro-inflammatory cytokines by gingival epithelial cells and oral keratinocyte,^31 , 32^ supporting its association with periodontal inflammation. Moreover, *F. alocis* was detected only in advanced stages of the disease among P patients. The low frequency of *Aa* (22%), particularly the JP2 highly leukotoxic clone (2.4%), in our periodontitis population agrees with the literature.^13 , 33 - 35^ Additionally, from six young adult patients who tested positive for the JP2 clone, five were of African origin and presented severe PD.^34^

We sought for potential synergistic or antagonistic relations among these periodontal species by looking at the observed/expected frequency of detection of distinct consortia in the three clinical conditions. Overall, the pathogens *Aa* and *D. pneumosintes* were key species among the consortia strongly associated with disease. For instance, *Aa* + *F. alocis* + *D. pneumosintes* were commonly detected in combination in periodontitis, but not in G or PH groups, whereas the consortium *Aa* + *P. gingivalis* + *D. pneumosintes* was significantly correlated with advanced disease. The *Aa* clone JP2 combined to *F. alocis* and *D. pneumosintes* was also associated with disease severity, although with a much lower strength. Further multivariate analysis including socio-demographic parameters previously related to disease showed that the consortium *Aa* + *P. gingivalis* + *D. pneumosintes* had increased age and African heritage as significant indicators of disease severity, reinforcing the multifactorial nature of PD.

A longitudinal study evaluating the onset of molar-incisor aggressive periodontitis in Hispanic/African American adolescents^36^ showed that the presence of the consortium *Aa* + *Streptococcus parasanguinis* + *F. alocis* was an indicative of further bone loss with a 89% sensitivity. Like our findings, only 3.9% of Aa-positive individuals had the JP2 type, with 89% being of African ancestry. Although *D. pneumosintes* failed to show an association with this consortium in their study, such species was among the few taxa that increased significantly in diseased adolescents compared to the individuals who remained healthy.^36^ Data on the mechanisms of interaction among these putative and candidate pathogens are hard to interpret, but evidence^37^ may support the association of the consortia *Aa* + *P. gingivalis* + *D. pneumosintes* , and to a lesser extent, of *Aa* JP2 + *F. alocis* + *D. pneumosintes* with PD severity. Ghayoumi, Chen, Slots^26^ (2002), for instance, found a significant association between *D. pneumosintes* and *T. forsythia* , but not between *D. pneumosintes* and *P. gingivalis* in subgingival samples of P patients. Similar to our findings, these species increased with patients’ age. Sha and Chen^38^ (2020) studied biofilm formation using various strains of *Aa* in co-culture with *P. gingivalis* and *D. pneumosintes* . They reported a synergistic relation between *Aa* + *D. pneumosintes* for most of the *Aa* strains evaluated, but failed to find significant differences between the amounts of single-species and dual-species *Aa* + *P. gingivalis* biofilms. In another biofilm study,^39^ a *P. gingivalis* strain with high biofilm-formation ability and proteolytic activity, presented a competitive advantage against *Aa* during biofilm formation. Although *D. pneumosintes* has been considered a relevant pathogen in oral infections for over 20 years, information on its putative virulence factors related to the pathogenesis of periodontitis is scarce.^40^ A recent systematic review suggested a relation of *Dialister* spp. with several types of cancer.^41^ One of their arguments is that metabolic end products of *Dialister* spp., such as acetate and lactate, may play a role in carcinogenesis. Yost, et al.^42^ (2018) reported a striking increase in the general expression of virulence genes in oral squamous cell carcinoma-associated microbiomes, which were enriched with *Fusobacterium, Johnsonella* , and *Dialister* . Nevertheless, the mechanisms by which species enhances or inhibits gene expression by other species in a consortia comprising *D. pneumosintes* needs to be further explored.

Regarding the candidate pathogen *F. alocis* , when evaluating the frequency of pairs of species in different clinical conditions, *Aa* JP2 clone correlated only with *F. alocis* in severe disease. The *Aa* strain was also frequently observed along with *F. alocis* in P samples, although with a lower phi coefficient. The high prevalence of *Aa* JP2 combined to *F. alocis* in subgingival biofilm from advanced forms of P (stages III/IV) compared to initial/moderate disease somehow agrees with the findings reported by Wang, et al.^43^ (2013). They investigated the community interactions of *F. alocis* with commensal and pathogenic periodontal species. While *Streptococcus gordonii* had an antagonistic effect on *F. alocis* growth, mutualistic growth was observed for a biofilm co-culture of *Aa* and the specific D-62D strain of *F. alocis* . Likewise, in co-culture with *P. gingivalis, F. alocis* D-62D strain showed increase adherence and invasion of epithelial cells, as well as an increase in biofilm formation.^44^ Thus, these relations and their impact on biofilm virulence during PD onset and progression may be driven at the strain or clone levels, and not limited to species.^38 , 39 , 43 , 44^ Our PCR method was not designed to identify specific strains or clones of these oral species except for *Aa* JP2.

An interesting finding was the potential antagonism found in *D. pneumosintes* and *P. gingivalis* or *F. alocis* in biofilm samples from G but not from P patients. In the non-destructive inflammatory state of gingivitis, *D. pneumosintes* seems to compete with either *P. gingivalis* or *F. alocis* , since they are more frequently detected single than paired ( *D. pneumosintes* + *P. gingivalis* or *D. pneumosintes* + *F. alocis* ). These results may be a consequence of the overall high prevalence of *F. alocis* and *P. gingivalis* and the low frequency of *D. pneumosintes* in G. On the other hand, *D. pneumosintes* is suppressed by other oral species at this initial stage. For example, Siqueira and Rôças^45^ (2003) also reported a dissociation between *D. pneumosintes* and *P. gingivalis* in samples from abscessed teeth and infected root canals with periradicular lesions. Shifts in the periodontal microenvironment from G to P may favor the overgrowth of this candidate pathogen, which is frequently associated with *Aa* + *P. gingivalis* or *F. alocis.* Accordingly, the combinations *D. pneumosintes* + *P. gingivalis* and *D. pneumosintes* + *F. alocis* revealed a strong association with deep periodontal pockets^46^ and sites with progressing periodontitis,^47^ respectively.

Despite the significant correlations between specific consortia and disease severity, our data must be considered within its methodology limitations. Although many individuals were analyzed, we targeted only four periodontal pathogens among several species that compose the subgingival biofilm. Moreover, our aim was not to evaluate the levels, viability, or virulence of these species and strains. Therefore, other pathogenic consortia correlated to disease severity may predominate in distinct populations presenting PD. Studying the mechanisms that occur in biofilm shifts from a healthy state to PD is not an easy task. Several distinct *in vitro* and *in vivo* approaches are needed to investigate the interactions among a wide range of species, even novel taxa not yet recognized. Nevertheless, our data reinforce the potential role of specific microbial communities in PD, indicating that the consortium comprising the putative pathogens *Aa* and *P. gingivalis* , and the candidate pathogen *D. pneumosintes,* associated with older age and African ancestry, increases the likelihood of severe periodontitis. Under the clinical point of view, this information may contribute to discriminate individuals with greater risk of having severe disease, and to develop a more personalized approach focusing on arresting disease onset and progression by targeting these key consortia.
